# Software Architecture of a Fog Computing Node for Industrial Internet of Things

**DOI:** 10.3390/s21113715

**Published:** 2021-05-26

**Authors:** Ioan Ungurean, Nicoleta Cristina Gaitan

**Affiliations:** 1 Faculty of Electrical Engineering and Computer Science, Stefan cel Mare University of Suceava, 720229 Suceava, Romania; 2 MANSiD Integrated Center, Stefan cel Mare University, 720229 Suceava, Romania

**Keywords:** industrial internet of things, fog computing, embedded system, fieldbus, system on chip

## Abstract

In the design and development process of fog computing solutions for the Industrial Internet of Things (IIoT), we need to take into consideration the characteristics of the industrial environment that must be met. These include low latency, predictability, response time, and operating with hard real-time compiling. A starting point may be the reference fog architecture released by the OpenFog Consortium (now part of the Industrial Internet Consortium), but it has a high abstraction level and does not define how to integrate the fieldbuses and devices into the fog system. Therefore, the biggest challenges in the design and implementation of fog solutions for IIoT is the diversity of fieldbuses and devices used in the industrial field and ensuring compliance with all constraints in terms of real-time compiling, low latency, and predictability. Thus, this paper proposes a solution for a fog node that addresses these issues and integrates industrial fieldbuses. For practical implementation, there are specialized systems on chips (SoCs) that provides support for real-time communication with the fieldbuses through specialized coprocessors and peripherals. In this paper, we describe the implementation of the fog node on a system based on Xilinx Zynq UltraScale+ MPSoC ZU3EG A484 SoC.

## 1. Introduction

The Internet of Things (IoT) [1] is an emerging concept that has begun to be used in all areas of activity. The purpose of this concept is to bring the physical things into the virtual environment where they can interact with each other. IoT devices acquire data from the environment and they transmit data to the Internet with or without local processing possibilities. Usually, these devices have a wireless connection to the Internet, and an important aspect is energy saving. IoT enables applications for smart building [2], smart transportation [3], smart city, smart healthcare [4], etc.

The Internet of Things (IoT) generates a large amount of data, with an estimated 79.4 zettabytes (ZB) being generated by 2025 [5]. Consequently, data transmission in the cloud can consume a large amount of bandwidth. In order to solve this issue, and to achieve a low bandwidth requirement, high security level, and low latency, Cisco Systems proposed a new paradigm [6] called fog computing. This concept brings cloud services such as data processing, storage, and aggregation to the edge of the network. With these services being closer to the devices that generate the data, a much shorter response time is obtained when offering these cloud services [6]. With the success of the IoT concept, it began to be used in the industrial environment, where it is called the Industrial Internet of Things (IIoT). The IIoT is a subset of the IoT, which consists of sensor networks (industrial fieldbuses), actuators, robots, machines, appliances, business processes, and personnel [7,8] in order to achieve an efficient and intelligent manufacturing process. With the development of the fog-computing concept for IoT, it has also started to be used for IIoT [8].

The main challenges in the development of fog computing systems [9] are represented by the software architecture of the fog computing systems, the integration of the network capabilities, the diversity of the network and devices that can be integrated into the fog systems, the security, the setup and control of the fog services by the end-users, the connections to the cloud, and the maintenance and customizing of the network and fog systems [10].

In this paper, we propose the software architecture and practical implementation method for a fog node that can be used for IIoT systems. This node is based on asymmetric processing to meet the real-time restrictions in the industrial environment and to decouple this component from hard real-time requirements from the application component. Each of these two components is executed on specialized co-processors. The proposed solution is based on the Xilinx Zynq UltraScale+ MPSoC ZU3EG A484 SoC that has Quad-core ARM^®^ Cortex™-A53 MPCore™, Dual-core ARM Cortex-R5 MPCore™. The practical implementation of the fog/edge node was performed on CANOpen fieldbus in two variants. In the first variant, the driver for the CANOpen fieldbus is implemented on the ARM Cortex A53 core under a Linux operating system. In the second variant, the ARM Cortex R5 core is used to deal with the communication on the CANOpen fieldbus.

The elements of originality and innovation are that it uses asymmetric multiprocessing to achieve real-time requirements and to separate the application layer from the sensing layer. In addition, the implementation involves the integration of several types of fieldbuses through a uniform method for fieldbus description.

This research paper is structured as follows: Section 2 presents some solutions for fog computing from the specialized literature and the middleware system used in the IoT and IIoT; Section 3 proposes a software architecture for a fog/edge node that can be used to build IIoT system by integrating the fieldbuses specific to the industrial field; Section 4 presents some discussions related the implementation of the fog/edge node using the Xilinx Zynq UltraScale+ MPSoC ZU3EG A484 SoC; The final conclusions are drawn in Section 5.

## 2. Related Works

This section presents some solutions for fog computing from the specialized literature and the middleware system [11,12] used in the IoT and IIoT.

We start with reference architectures for fog computing. A reference architecture is an architecture with a high degree of abstraction used to identify the main component blocks and the main challenges for the design, development, and implementation of a practical solution, without providing implementation details.

To come to the aid of the developers of fog computing solutions, the OpenFog Consortium (now part of the Industrial Internet Consortium) defined a reference architecture for fog computing [13]. The architecture integrates open technologies and approaches issues related to latency, bandwidth, and distributed coordination. The architecture uses intelligent endpoints, reliable networks, and secure data flows between the cloud, endpoints, and services. The pillars of the OpenFog architecture are security, scalability, openness, autonomy, reliability, availability, serviceability, agility, hierarchy, and programmability [13]. This reference architecture has a high degree of abstraction and it does not define how the things from the real environment are integrated into a practical solution, which is at the discretion of the developers of solutions for IoT or IIoT based on fog computing.

There are many challenges involved in the open protocols and architectures of the fog computing for the end-users, many of which are related to the security of resources and the decrease in energy consumption. Thus, in [14], a reference architecture Ii presented consisting of 5 layers:

1. The IoT application layer;
2. The management of resources defined by software layer;
3. The resources and cloud services layer (via a computing platform that manages IoT resources and applications);
4. The network and access to gateways (which are connected through the network layer and which provide connectivity services to edge systems);
5. The sensors and edge devices layer.

A fog’s software architecture description is also presented in another reference [15] that focuses on various technological components of fog computing (hierarchical and distributed platforms for providing services). It also detailed how fog completes and expands the cloud computer, key aspects of the fog computing, as well as various cases that motivated the need for fog, and emphasizing the relevance of fog for several verticals in the IoT and Big Data space. A hierarchically distributed architecture has also been proposed that extends from the edge of the network to the fog core, including how to add a large number of distributed sources.

In [16], the authors proposed a new reference architecture for fog computing based on the extension of the SDN [17] and NFV [18] reference architecture by manipulating network resources (e.g., overcoming a dramatic increase in user traffic), cloud, and fog. They also designed a distributed SDN system for the implementation and management of VNFCs over the network and to meet the stringent requirements of fog computing.

Some solutions for IIoT fog computing found in the literature are presented as follows. The paper [19] contains a study related to the security implications of fog computing on the IoT. The authors conclude that although the integration of fog computing in IoT seems to be non-trivial and complicated, the benefits outweigh the costs. In [20], the authors study the Industrial IoT control applications that are virtualized as software tasks running on a fog computing platform that brings computing and deterministic communication closer to the edge of the network. They propose a simulated annealing-based metaheuristic to determine the mapping of the tasks and a schedule table of their activation in order to maximize the quality-of-control for the control tasks and meet the timing requirements for all tasks.

A fog industrial big data integration and sharing (fog-IBDIS) platform is proposed in [21] in order to protect raw data privacy and enable data integration and sharing. They present a case study that illustrates the implementation of fog-IBDIS, which ensures raw data security by deploying the analysis tasks executed by the data generators.

An optimal data scheduling policy with multiple communication channels to minimize real-time processing delay and increase the stability of the system is proposed in [22]. A series of experiments are presented in order to evaluate behaviors with three different scheduling policies.

In the specialized literature, there are a lot of references, such as [23,24,25,26], related to the use of fog computing related to the IoT or IIoT, but they do not deal with the integration of objects connected to different sensor networks.

In [27], the author proposes a cognitive Cogni-IoT platform that is used for an industrial fog computing application and requires distributed intelligence that the fog computing paradigm promises to apply to the edge. By using a predictive maintenance (PdM) algorithm based on PdM-as-a-service for IoT applications and considering multiple cognitive agents (i.e., classifiers), he solved a common optimization problem and provided the best prediction for machine maintenance that could still work efficiently. An interesting definition of and presentation on the Industrial Internet of Things (IIoT) is made in [28]. The authors highlight the latest IIoT research efforts in three areas, including IIoT architectures and frameworks, communication protocols and data management techniques, and various IIoT-related enabling technologies. They identified major IIoT research challenges that include efficient data management schemes, trust in IIoT systems, collaborations between heterogeneous IIoT systems, specific operating systems and public safety in IIoT, robust and flexible big data analytical technologies, coexistence of wireless technologies and IIoT protocols, and allowing decentralization of the edge. Detailed research on the latest studies on security, architecture, consumption, and latency that fog computing (FC) can present at the industrial level was conducted by Caiza et al. in [29]. One of the observations made by the authors was that most researches have focused on the development of fog computing for applications where latency and security are not an issue. They proposed that a system have different methodologies to obtain better performance. The development of a fog service orchestrator (Q-FSO) for large-scale IIoT application provisioning in a fog computing environment was proposed by Jen-Sheng Tsai et al. in [30], addressing the issue of scalability and ensuring a quality of service (QoS) guarantee. The authors proposed two heuristic algorithms, incremental similarity matching (ISM) and greedy multiple matching (GMM), for efficient allocation of IIoT services, concluding that both algorithms outperform current QoS solutions, heterogeneity, scalability, dynamism, and interoperability.

In IoT and IIoT solutions, an important feature is represented by the security capabilities. These capabilities are important for the protection of critical data, especially in the industrial field. IIoT security has been analyzed in detail in the literature and several solutions have been proposed. For example, in [31], the authors propose a solution based on a physical unclonable function (PUF) for key-sharing in which shared keys are physically generated. This solution has the advantage that it uses few hardware and computing resources and can be used on devices with resource constraints. In [32], the authors analyze lightweight security solutions based on a physical unclonable function and present machine learning (ML)-based modeling attacks to break such authentication.

In the process of designing and developing fog-computing solutions for IIoT, we need to take into account the characteristics of the industrial environment that must be met. These include low latency, predictability, response time, and operation with hard real-time compiling. We can take for our starting point the reference fog architecture released by the OpenFog consortium. This, however, has a high abstraction level and does not define how to integrate the fieldbuses and devices into the fog system.

We can conclude that the biggest challenges in the design and implementation of fog solutions for IIoT are the diversity of fieldbuses and devices used in the industrial field, as well as complying with all constraints in terms of real-time compiling, low latency, and predictability.

Fog and edge computing are new concepts integrated into the Internet of Things in order to process data closer to where they are generated before being transferred into the cloud. Fog computing uses interconnection between nodes while edge computing is performed on isolated nodes [33].

In [34], a comprehensive study is performed on the communication protocols used for IoT. The main middleware protocols identified are message queuing telemetry transport (MQTT) [35], advanced message queuing protocol (AMQP) [36], constrained application protocol (CoAP) [37], extensible messaging and presence protocol (XMPP) [38], data distribution service (DDS) [39], and OPC UA [40]. From these middleware protocols, DDS stands out because it can support real-time communication and data transmission with the publisher-subscriber paradigm based on a decentralized data model. It also supports 23 QoS levels as opposed to AMQT and MQTT with 3 levels. The disadvantage is the weak support for low power consumption. MQTT stands out because it is supported by all cloud platforms for IoT while DDS is not supported by these cloud systems. We can conclude that the DDS middleware system can be used to interconnect fog/edge nodes and the MQTT middleware system can be used for secure connections to cloud servers.

## 3. The Software Architecture of the Proposed Fog/Edge Node

This paper proposes a software architecture and a method for the practical implementation of a fog/edge computing node that can monitor and control in real-time a set of fieldbuses and locally process the data at the edge of the local network before transmitting it to a cloud server. It also provides a software middleware interface for accessing data acquired from fieldbuses in order to build and develop IIoT applications by interconnection of the fog/edge nodes and others software modules. Furthermore, the fog node can use secure connections to cloud servers for long-term data storage and analysis using the computing power provided by the cloud. The interface of the data acquired from fieldbuses is provided through a middleware system based on the DDS standard. Furthermore, the proposed architecture is compatible with the Node View of the reference architecture proposed by OpenFog Consortium, this being argued in the current section.

**Figure 1:**
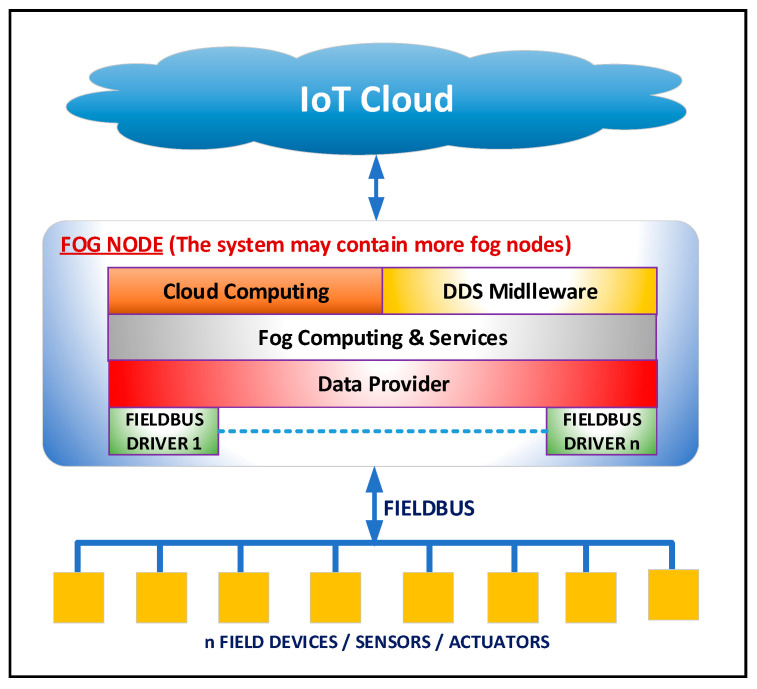
The general architecture of the proposed fog node.

The software architecture of the proposed fog/edge node is presented in Figure 1. It is based on the model presented in [41], which has been extend in order to be implemented in reality. From a software point of view, in [41] a fog node is proposed that is organized into two layers. This organization is detailed and is extended to four layers as shown in Figure 1. In this paper, the emphasis is on the driver layer where a CANOpen fieldbus is integrated. Basically, this paper proposes an implementation of the IIoT concept presented in [41].

From the software point of view, the fog node is organized into four layers: fieldbus drivers, data provider, fog computing & services, and middleware (Cloud Computing & DDS middleware). Between these layers, there are defined standard software interfaces that allow the independent design and execution of the software modules associated with each layer.

### 3.1. The Fieldbus Drivers Layer

From the software point of view, the specific driver for each fieldbus integrated into the fog node is designed and implemented (see Figure 1). The drivers implement fieldbus-specific communication operations using an interface, an adapter, or a port/peripheral provided by the SoC system on which it is running. Basically, it implements the complete stack for a fieldbus-specific master device (depending on the specifications of the fieldbus). At this level, the address space for the fieldbus is built at a minimum level as it is described in [41], and each object from the address space will have associated the operations that must be performed when reading the object-associated values from the fieldbus or for transmitting the associated value to the fieldbus. The fieldbus driver has access to the buffer memory allocated at the top layer in order to store the acquired data from the fieldbus and to retrieve data that is transmitted to the network. More details on implementing a driver are presented in Section 5.

### 3.2. The Data Provider Layer

The data provider layer builds the address space of the fog node (see Figure 1). This layer defines the objects that have correspondents in the devices connected to the fieldbuses. The build and definition of the address space are performed in the same way as the one presented in [41]. The address space consists of a list of fieldbuses, where each fieldbus has a list of devices and each device has a list of objects. Each object can have several sub-objects, each sub-object being characterized by several attributes such as: value, data type, timestamp, quality, and type of access. The data provider allocates a buffer memory that is used to store data associated with objects. The fieldbus drivers layer updates the buffer memory with the data acquired from the fieldbuses, and all requests from the fog computing & services layer are processed through this buffer.

### 3.3. The Fog Computing & Services Layer

The Fog Computing & Services layer contains the fog services provided by the fog node (see Figure 1). These services include storage, data aggregation, and other specific services, as well as various services that allow the design and development of different applications specific to the industrial field such as supervisory control and data acquisition (SCADA) systems. At this layer, the user establishes the rules used for data aggregation and processing and can setup the data acquired from the fieldbuses (data from sensors or transducers), data that are transmitted on the fieldbuses (e.g., commands to actuators), data that are published through the DDS middleware [42], and data to which it subscribes in the available DDS topics.

As described in [41], in order to enable data processing and storage services at this layer, different objects can be instantiated that interact with each other through a virtual environment by exchanging data. Moreover, this layer can create virtual objects that connect to other objects in the virtual environment. These virtual objects perform various mathematical operations with the acquired values and publish the new data in the virtual environment.

### 3.4. The Middleware Layer

**Figure 2:**
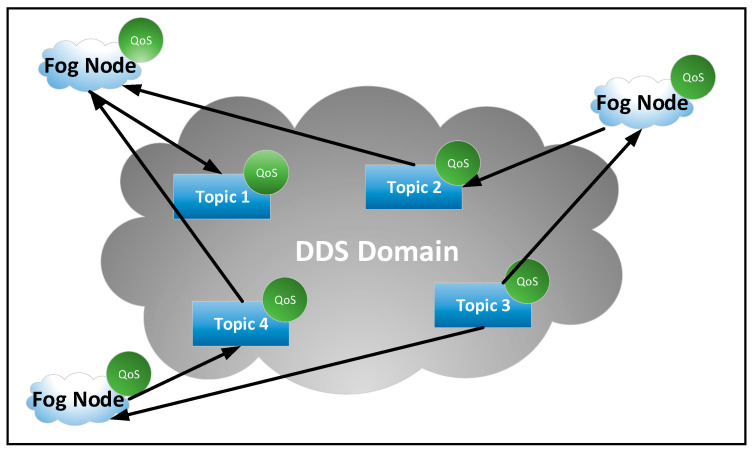
An example of interconnections between fog nodes via data distribution service (DDS) middleware.

The Middleware layer (see Figure 1) is the software part that publishes data in DDS topics and that subscribes to various data published in DDS topics (see Figure 2). This layer can also provide secure connections to cloud servers in order to access specific centralized cloud services.

The fog node is compliant with the reference architecture for fog computing released by OpenFog Consortium, and it uses the DDS protocol to interconnect the fog nodes. The fog nodes exchange data with each other via the DDS protocol [42]. The retrieved data can be used to process data locally. This mechanism allows things from the industrial environments to be interconnected via fog nodes to activate the IIoT concept. The interconnection of fog nodes is shown in Figure 2. Each node can define DDS topics with different QoS properties where it publishes data. Other nodes can be subscribed to these topics to retrieve data and use them locally to aggregate data. Various mathematical functions can be applied over local and retrieve data to obtain other new data. This new data can be published in DDS topics or transmitted on through cloud servers. Regarding the security of fog nodes, it is ensured by implementing DDS security specifications that allow the activation of different security levels. In addition, all configurations of fog nodes are permitted only after authentication with credentials that ensure these rights.

Through the fog node, we can set deadlines that are very important in the hard real-time systems used in the industrial field, data from which are processed by the fog node. The aim of the fog node is to support IIoT by meeting the requirements that are specific to the industrial field. So far, the general architecture of the fog node has been presented, but the hardware platform is also very important. The design and development of the fog node are performed from the software and hardware perspective by using development kits based on modern SoCs that allow data acquisition from these fieldbuses. The focus is on the preservation of the characteristics specific to the industrial environment.

### 3.5. Selecting an SoC Based System for Practical Implementation

The most important layer is the fieldbus drivers, because in this layer the specific requirements of the industrial environment must be met. For this reason, it is very important which hardware platform is selected. A solution can be the use of modern SoCs or multicore microprocessors that have specialized cores for real-time tasks.

Currently, the main microcontroller manufactures developed SoC solutions that have one or more ARM Cortex Ax cores and one or more specialized cores based on ARM Cortex Mx or other architecture for developing real-time applications. Thus, it is possible to perform a decoupling of the real-time part of the application, which involves the processing of data at the edge of the network and the cloud services provided by a fog node. Examples of these types of SoCs are i.MX 7 series (Cortex A7 & Cortex M4) and i.MX 8M (Cortex A53 & Cortex M4F) from NXP, STM32MP1 microprocessor series from ST Microelectronics (Cortex A7 & Cortex M4), Sitara™ AM4x (Cortex A9 & PRU-ICSS for industrial communication) and Sitara™ AM6x (Cortex-A53 & Cortex-R5F) from Texas Instruments, and Zynq UltraScale+ MPSoC (Cortex-A53 & Cortex-R5F) from Xilinx. These SoC solutions provide application cores that can be used with a complex operating system (such as Linux Embedded, Windows 10, and Android) to execute the tasks/processes for the application layer that don’t have hard real-time requirements and cores for hard real-time tasks, which can use real-time operating systems (RTOS). These solutions can be used to design and develop a fog node for the devices from industrial environments with specific characteristics such as hard real-time compiling and low latency. These requirements can be achieved by using specialized cores that handle the communication throughout fieldbuses and imply the use of asymmetric and heterogeneous multiprocessing.

In this paper, we propose the development of an edge/fog node based on the Xilinx Zynq UltraScale+ MPSoC ZU3EG A484 SoC that has Quad-core ARM^®^ Cortex-A53 MPCore™, Dual-core ARM Cortex-R5 MPCore™, and communication peripherals such as CAN, UART, SPI, I2C, and Tri-mode Gigabit Ethernet. These characteristics make it a candidate for the decoupling of edge/fog computing nodes for the industrial environment. Thus, the architecture proposed is focused on this SoC.

## 4. Implementation of the Proposed Architecture & Discussions

The proposed solution suggests a new approach because it allows the decoupling of the application component from the real-time component specific to the industrial field. The fog nodes interact and exchange data between them via DDS middleware. This middleware allows the definition of QoS parameters to obtain low latency and real-time performance for communication. To achieve the proposed objectives, we used a modern SoC (Zynq UltraScale+ MPSoC ZU3EG A484) that have specialized cores for real-time tasks.

For the fieldbus drivers layer, we defined a standard software interface used by the fog computing & services layer to instantiate these drivers and to exchange data with the devices connected to the fieldbuses. In addition, the fog computing & services layer has a standard interface that it is used by the cloud and middleware layer. After defining these specifications, we proceed to the software design of the fog node.

For this implementation, we used the Ultra96-V2 Zynq UltraScale+ ZU3EG Single Board Computer using a Linux operating system. Except for the drivers for fieldbuses, the other software components can be designed and developed independently of the hardware platform. In contrast, fieldbus drivers are specific to the SoC chosen, depending on the specialized cores, the way of communication between the application cores and specialized cores, and the peripherals used for connection to the fieldbuses.

The biggest challenge is the design and development of these fieldbus drivers. The Zynq UltraScale+ MPSoC provides several communication peripherals such as SPI, I2C, UART, CAN, Ethernet, and USB. These peripherals can be used to connect directly to the fieldbuses or by using adapters (e.g., UART to fieldbus or I2C to fieldbus).

In this paper, we proposed an implementation of a driver for the CANOpen network based on a CAN port provided by Zynq UltraScale+ MPSoC. The CAN ports can be accessed both from the ARM Cortex A53 cores that use a Linux distribution as the operating system and from the ARM Cortex R5 that can use a real-time operating system.

In the first instance, we describe the implementation of the CANOpen driver on the ARM Cortex A53 cores, after which some of the functionalities are transferred to the ARM Cortex R5 cores. Within the driver, an acquisition cycle is divided into several time slots of equal size. One or more slots may be associated with an object that is periodically updated on the CANOpen fieldbus using service data object (SDO) requests. In addition, the CANOpen fieldbus has defined process data object (PDO) objects that are periodically transmitted on the fieldbus. For this reason, the driver needs access to the CANOpen configuration file to be able to interpret this data and to connect to the objects defined for the CANOpen fieldbus.

The CANOpen objects can associate one or more slots from the acquisition cycle depending on the size of the related data. These slots can be used to update the objects using read or write requests sent to the CANOpen fieldbus. The dimensions of the acquisition cycle and the slots are set in the setup part of the fog node depending on the communication speed of the CAN network. One or more slots may remain un-associated / free to be used to handle requests for CANOpen objects that are not included in the acquisition cycle.

**Figure 3:**
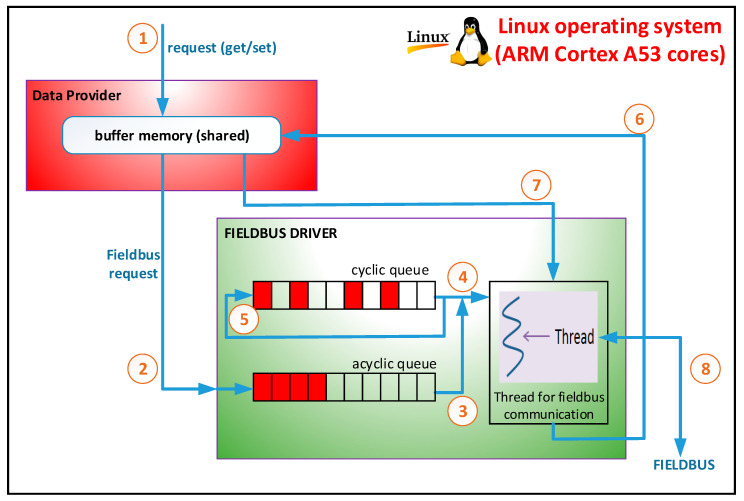
The operation diagram for a CANOpen fieldbus driver.

Figure 3 presents an operation diagram for a fieldbus driver when general-purpose CPUs (such as the ARM Cortex A53 cores) are used and specialized coprocessors are not. In this case, all modules of the fog node are executed on the Linux operating system that runs on the ARM Cortex A53 cores. The data provider receives read and write requests for the defined objects (see (1) in Figure 3). If data provider receives a write request then the data is updated in the buffer memory and a write request is sent to the fieldbus driver (see (2) in Figure 3).

The fieldbus driver checks if the object is in the acquisition cycle (cyclic queue), and if it is not it will be inserted into the queue with the asynchronous request (acyclic queue). If data provider receives a read request for an object, the request is served immediately from the buffer memory, and a request is sent to the driver (see (2) in Figure 3). The driver checks if the object is in the acquisition cycle, then the object will be inserted in the queue with the asynchronous request (acyclic queue). It should be noted that the memory buffer from data provider is periodically updated for the objects from the acquisition cycle. For the objects that are not in the acquisition cycle, the memory buffer is updated when a read operation has been performed. When a new read request is performed for the object, the buffer memory will contain updated data.

**Figure 4:**
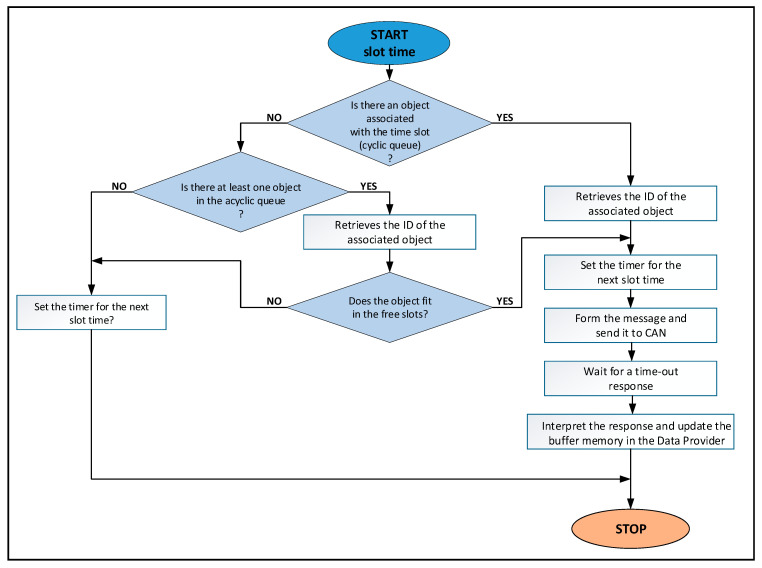
The activity diagram for the CANOpen fieldbus driver.

As can be seen in Figure 3, within the fieldbus driver there are two queues. A queue is associated with the acquisition cycle, with one item for each slot (cyclic queue). The second queue contains requests for objects that are not included in the acquisition cycle (acyclic queue). The driver has a thread that runs periodically at the beginning of each slot. This thread extracts an item from the queue associated with the acquisition cycle. If this slot is associated with an object, then the request for that object will be processed (see (4) and (5) in Figure 3). If the slot is not associated with an object then an item is removed from the queue with asynchronous requests and the request for that object is processed (see (3) in Figure 3). This operation requires communication on the CANOpen fieldbus (see (8) in Figure 3). The result of the communication operations on the CANOpen fieldbus is sent to the buffer memory (see (6) from Figure 3). Data that is transmitted on the fieldbus can be retrieved from the buffer memory (see (7) from Figure 3). The buffer memory is protected by a semaphore to achieve mutual exclusion when it is accessed. Figure 4 presents the activity diagram of the thread that is executed at the beginning of each slot. Algorithm 1 presents the pseudocode for the activity diagram from Figure 4. If the object has associated two or more slots then the time-out and the next activation of the thread are adjusted accordingly.
**Algorithm 1:** SlotTime.1:**if** there is an object associated with the timeslot (cyclic queue) **then**2:  Retrieves the ID of the associated object3:**else**4:   **if** there is at least one object in the acyclic queue? **then**5:     Retrieves the ID of the associated object6:     **if** the object fits in the free slots **then**7:       **do noting**8:     **else** Set the timer for the next slot time9:       **return**10:     **endif**11:   **else** Set the timer for the next slot time12:     **return**13:   **end if**14:**end if**15:Set the timer for the next slot time16:Builds the message and send it to CAN17:Wait for a time-out response18:Interpret the response and update the buffer memory in the Data Provider19:**return**

One disadvantage of the proposed solution is that the communication with the CAN network is handled by a Linux thread that is not executed in real-time. The Linux operating system is not a hard real-time operating system but a soft real-time operating system (best effort). To handle the communication in hard real-time, the communication part with the CANOpen fieldbus can be executed on an ARM Cortex R5 core provided by Zynq UltraScale+ MPSoC. In this case, the fieldbus driver has a module that is executed on the application core under Linux and a module that is executed on specialized cores, encapsulating the communication between the heterogeneous cores and the asymmetric multiprocessing part.

The software module from the driver that is executed by the ARM Cortex A53 cores under Linux hides the specific characteristics of the fieldbus and the way of connecting to the fieldbus. It also encapsulates the communication part with the ARM Cortex R5 core, which executes the software that implements the specific protocol stack in compliance with real-time requirements.

These two components communicate with each other through messages, a mechanism provided by Linux to be able to communicate with specialized cores. The software on the specialized core uses an RTOS and implements the CANOpen stack. This software module can respect the specific real-time requirements. The module from the ARM Cortex A53 core works like a master that sends requests to the module from the ARM Cortex R5 core. The requests can be of two types: configuration requests and process requests through which the data are read or send to the fieldbus. Besides, the module from ARM Cortex R5 core can send notifications in case of detecting different events on the CANOpen fieldbus.

The shared memory communication is used to transmit data between two modules of the CANOpen driver that are executed on ARM Cortex A53 and ARM Cortex R5. In this case, it needs to invalidate the cache before reading (or the shared DRAM memory region can be configured as non-cacheable).

**Figure 5:**
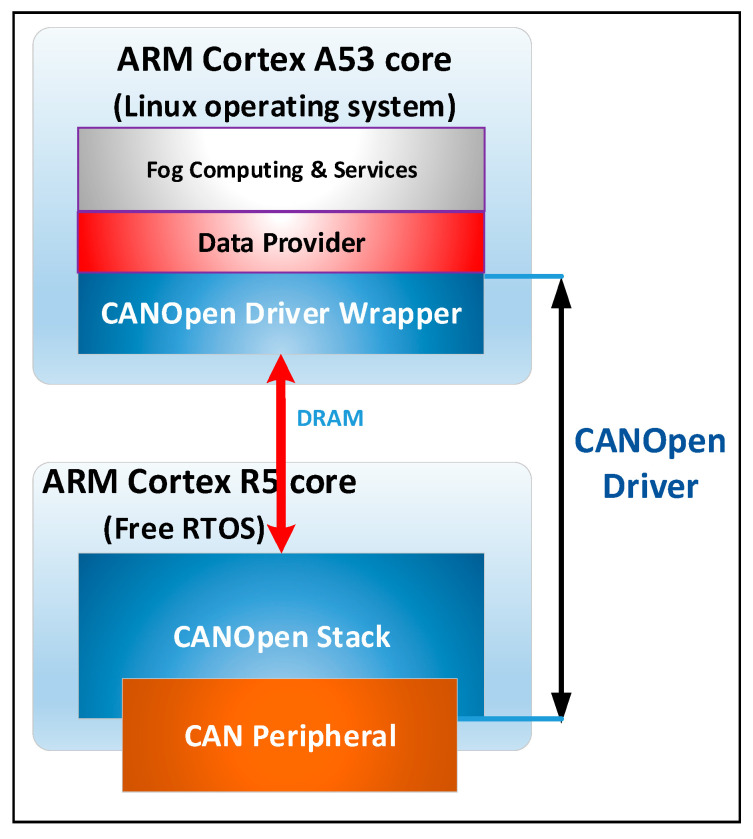
The software architecture of the CANOpen fieldbus driver.

Figure 5 presents the software architecture of the driver for CANOpen fieldbus. It has a software component on the ARM Cortex A53 core that is executed under the Linux environment and a software component that is executed by the ARM Cortex R5 core. On the specialized core, the software component that implements the CANOpen stack is based on the CAN peripheral provided by the SoC system.

**Figure 6:**
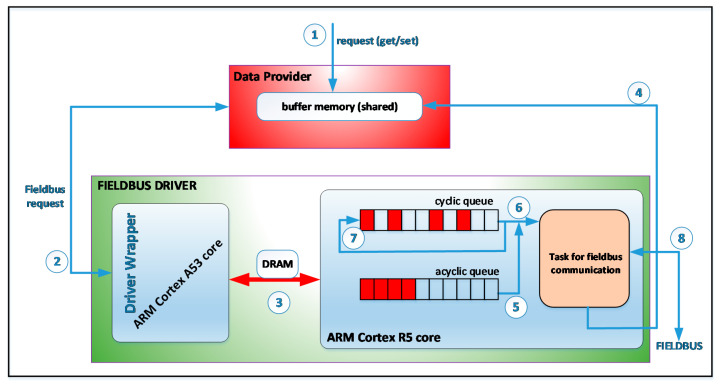
The operation diagram for a CANOpen fieldbus driver when a specialized core is used.

In Figure 6, we present an operation diagram for the CANOpen driver when the communication is handled in real-time on the ARM Cortex R5 core. In the figure, we can see the wrapper that receives the requests from the data provider (see (2) from Figure 6) that are received from de upper layer (see (1) from Figure 6). These requests are transmitted to the ARM Cortex R5 core through shared memory and a signal send to the A5 core (see (3) in Figure 6). The ARM Cortex R5 core executes a task at the beginning of each slot, and it implements the activity diagram from Figure 4. It handles the implementation of the acquisition cycle (see (6) and (7) in Figure 6), the requests for the objects that are not in the acquisition cycle (see (5) in Figure 6), and the communication on the CANOpen fieldbus (see (8) in Figure 6), and it updates the buffer memory from data provider (see (4) in Figure 6). By now, we have presented the implementation of a fieldbus driver for CANOpen. Furthermore, the SoC system provides communication interfaces for Ethernet, UART, SPI, I2C, USB that can be used to develop drivers for fieldbuses such as MODBUS, Ethercat, Profibus, Profinet, and so on. The software module from the ARM Cortex R5 core must implement all the functionality of the fieldbus. It receives, via messages, requests for data from the fieldbus or data that must be sent on the fieldbus. It must be specified that PLCs or devices that read data from sensors or transducers or various specific execution elements can be connected to the fieldbuses.

**Figure 7:**
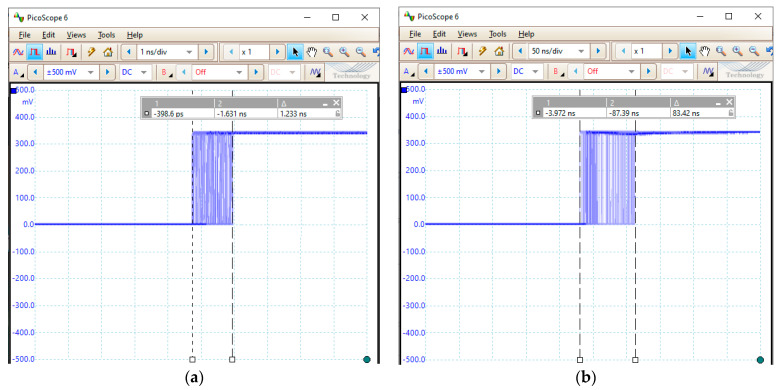
The jitter for the SYNC signal obtained for implementation on the ARM Cortex A53 core (**a**) and on an ARM Cortex R5 core (**b**).

The fieldbus driver for CANOpen operates as a master device within a CanOpen fieldbus, implementing the CANOpen Master Protocol Stack. One of the services you need to provide is to generate the Sync signal. It generates the sync message depending on the communication cycle period. For real time systems, it is important that this jitter is as small as possible. As can be seen in Figure 7, for the implementation of a cycle period of 400 ms on ARM Cortex A53 cores with Linux as the operating system, the SYNC is generated with a jitter of about 83 ns, and for the implementation on an ARM Cortex R5 core with FreeRTOS as the operating system, a jitter is obtained of approx. 1.233 ns. In order to obtain on the oscilloscope the graphics from Figure 7, the SYNC signal was configured to be generated every 100 ms, and before generating the SYNC signal, the line port that is connected to the oscilloscope probe is switched from 0 logic to 1 logic (which is switched back to 0 logic at the middle of the 100 ms period so that it can be prepared for the next generation of the SYNC signal). By setting the oscilloscope trigger on the rising edge, the images shown in Figure 7 are generated.

## 5. Conclusions

In this paper, we propose a design and a method of development for a fog node that is performed in terms of both software and hardware using development kits based on modern microcontrollers that allow data acquisitions through the fieldbuses. The fog node is designed for the Xilinx Zynq UltraScale+ MPSoC ZU3EG SoC, and uses the ARM Cortex R4 core for the execution of the driver to comply with the real-time requirements specific to the industrial field. From the functional and software perspective, the fog node is organized on four layers: fieldbus drivers, data provider, fog computing & services, and middleware. The biggest challenge is the design and development of the drivers for fieldbuses because this implies the use of asymmetric processing. The practical implementation of the fog/edge node was performed for CANOpen fieldbus in two variants. In the first variant, the driver for the CANOpen fieldbus is implemented on the ARM Cortex A53 core under a Linux operating system. In the second variant, the ARM Cortex R5 core is used to deal with the communication on the CANOpen fieldbus. By using the specialized processor, the jitter for the SYNC signal (specific to the CANOpen fieldbus) decreased from 83 ns to 1.233 ns.

## Data Availability

Data sharing is not applicable.
